# The development of the amnion in mice and other amniotes

**DOI:** 10.1098/rstb.2021.0258

**Published:** 2022-12-05

**Authors:** Susana M. Chuva de Sousa Lopes, Bernard A. J. Roelen, Kirstie A. Lawson, An Zwijsen

**Affiliations:** ^1^ Department of Anatomy and Embryology, Leiden University Medical Centre, Einthovenweg 20, 2333 ZC Leiden, The Netherlands; ^2^ Ghent-Fertility and Stem Cell Team (G-FAST), Department of Reproductive Medicine, Ghent University Hospital, Corneel Heymanslaan 10, 9000 Ghent, Belgium; ^3^ Anatomy and Physiology, Department of Clinical Sciences, Faculty of Veterinary Medicine, Utrecht University, Yalelaan 1, 3584CL Utrecht, The Netherlands; ^4^ Department of Biosciences, Biotechnologies & Biopharmaceutics, University of Bari Aldo Moro, Bari, Italy; ^5^ MRC Human Genetics Unit, IGC, University of Edinburgh, Crewe Road South, Edinburgh EH4 2XU, UK; ^6^ Cardiovascular Sciences, Center for Molecular and Vascular Biology, KU Leuven, Herestraat 49 box 911, 3000 Leuven, Belgium

**Keywords:** extraembryonic membranes, amnion, amniochorionic fold, mouse gastrulation, amniotes, clonal analysis

## Abstract

The amnion is an extraembryonic tissue that evolutionarily allowed embryos of all amniotes to develop in a transient and local aquatic environment. Despite the importance of this tissue, very little is known about its formation and its molecular characteristics. In this review, we have compared the basic organization of the extraembryonic membranes in amniotes and describe the two types of amniogenesis, folding and cavitation. We then zoom in on the atypical development of the amnion in mice that occurs via the formation of a single posterior amniochorionic fold. Moreover, we consolidate lineage tracing data to better understand the spatial and temporal origin of the progenitors of amniotic ectoderm, and visualize the behaviour of their descendants in the extraembryonic–embryonic junctional region. This analysis provides new insight on amnion development and expansion. Finally, using an online-available dataset of single-cell transcriptomics during the gastrulation period in mice, we provide bioinformatic analysis of the molecular signature of amniotic ectoderm and amniotic mesoderm. The amnion is a tissue with unique biomechanical properties that deserves to be better understood.

This article is part of the theme issue ‘Extraembryonic tissues: exploring concepts, definitions and functions across the animal kingdom’.

## 1. The amniote embryo as a Russian doll: innovations to colonize the terrestrial environment

**Figure 1.**
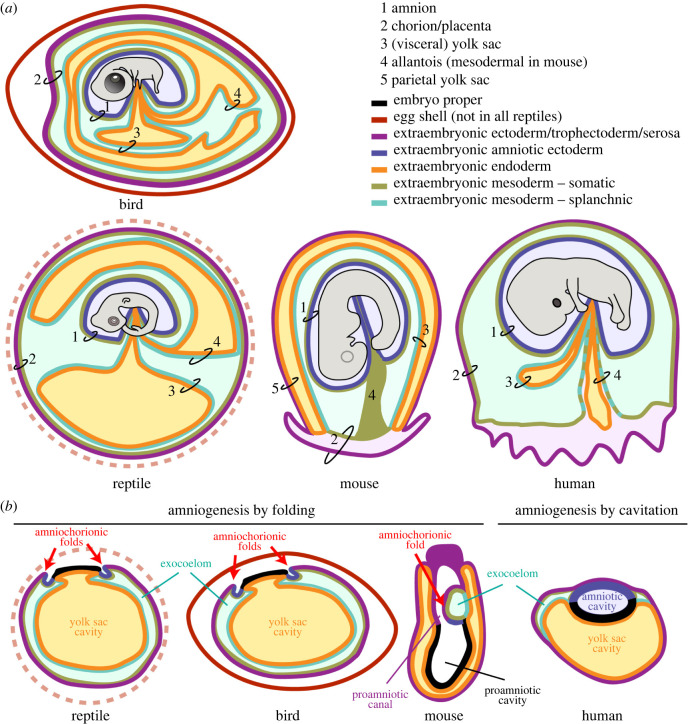
Extraembryonic fetal membranes in amniotes. (*a*) Schematic representation, not to scale, of the extraembryonic membranes in a typical bird, reptile, mouse and human. Indicated are (1) amnion, (2) chorion/placenta, (3) (visceral) yolk sac, (4) allantois (mesodermal in the mouse) and (5) parietal yolk sac (present in the mouse). The different tissues are colour coded as indicated. Modified from [1] and [2]. (*b*) Schematic representation, not to scale, of a reptile, bird, mouse and human embryo, at the time the amniotic ectoderm emerges. In reptiles and birds, amniogenesis initiates by the generation of an anterior and a posterior amniochorionic fold (red arrows) that envelop the embryo and meet dorsally at the midline. Mice generate one single posterior amniochorionic fold (red arrow) that expands laterally meeting anteriorly of the proamniotic canal. In humans, the amniotic ectoderm is formed by cavitation of the internalized inner cell mass, generating the epiblast and the amniotic ectoderm as well as the newly formed amniotic cavity early during development.

The evolution of several types of extraembryonic membranes, such as the amnion, the yolk sac, the chorion and the allantois (figure 1), has enabled a group of vertebrates to reproduce independently of aquatic surroundings and therefore to exploit and colonize the terrestrial environment of the earth. These different adaptations guaranteed a supply of nutrients and gas exchange to the developing embryo, while preventing dehydration of the developing embryo. In addition, the transition to the land was also supported by further innovations such as internal fertilization, the use of cornified epithelia in hard skin appendages (including scales, claws, feathers and hair) and ways of feeding that led to specific changes in the skull anatomy [3,4].

The clade Amniota includes all descendants of the ancestral mammals, lepidosaurs, turtles, crocodylians and birds. In the first amniotes that evolved, around 340 million years ago during the Carboniferous Period [5], embryos were contained within an egg with a more or less calcified eggshell (oviparous, a system still present in various reptiles, all birds and the monotreme mammals). In the course of evolution, mechanisms developed allowing the embryo to hatch from eggs inside the body of the mother (ovo-viviparous, as observed in various reptiles) or where the embryo develops entirely within the mother, without the formation of an eggshell (viviparous; various reptiles, all marsupial and eutherian mammals) [1]. In reptiles that have adopted viviparity, the fetal membranes need an organization that facilitates fetomaternal exchange, similar to the eutherian placenta.

In most amniotes (but not in mice), the outer extraembryonic membrane, known as the chorion (or serosa in birds and reptiles), interfaces with either the environment or the mother. The chorion consists of trophectoderm/extraembryonic ectoderm and extraembryonic (somatic) mesoderm (figure 1*a*). Notably, in the mouse, the ‘chorion’ is the name given to the structure that gives rise to the placental plate and does not surround the embryo (figure 1*a*); the membrane that interfaces with the mother in mice is the parietal yolk sac, consisting of trophectoderm and parietal endoderm [6]. However, mice should not be singled out as an exception as mammals show diversity in placentation strategies, and the parietal yolk sac—a membrane with trophoblast on the outside and primitive endoderm/hypoblast on the inside—is commonly observed in marsupials and other taxa, particularly in those showing an inverted yolk sac [7,8].

The yolk sac, composed of visceral endoderm/hypoblast and (splanchnic) extraembryonic mesoderm, encapsulates any existent maternally deposited yolk and typically develops primitive blood cells in the so-called blood islands that establish a primitive vascular plexus. The yolk sac is responsible, at least during early development, for metabolizing and transporting nutrients to the embryo [9]. In the mouse, the visceral yolk sac ends up encapsulating the amnion and the embryo [10] (figure 1*a*).

The allantois stores waste products but is rather vestigial in eutherians, whereas in egg-laying amniotes, the chorion and the allantois are vital and primarily needed for gas exchange across the eggshell. In birds and reptiles, the allantois is formed by extraembryonic endoderm and shapes into a fluid-filled vesicle, covered by extraembryonic (splanchnic) mesoderm (figure 1*a*). There, the extraembryonic mesoderm of the allantois and the chorion eventually fuse to form a chorioallantoic membrane, facilitating gas exchange and electrolyte reabsorption from the allantoic cavity [1]. In mice, the allantois is a solid structure of extraembryonic mesoderm that extends and fuses to the chorion to become the umbilical cord (figure 1*a*), and, in humans, the allantois (endoderm and extraembryonic mesoderm) is incorporated into the umbilical cord.

The amnion is typically formed by two cell layers: an outer layer of squamous extraembryonic amniotic (somatic) mesoderm and an inner layer of squamous amniotic ectoderm, with a central basement membrane. Both layers are derived from the embryo proper (epiblast) and not from the trophectoderm/extraembryonic ectoderm and, importantly, the layer of the amnion facing the embryo, the amniotic ectoderm, is always non-adhesive to the (surface) ectoderm of the developing embryo. In primate embryos, the cells of the hypoblast, in addition to forming the yolk sac endoderm, also seem to form a mesh-like structure that has been referred to as extraembryonic mesoderm or extraembryonic mesenchyme. These extraembryonic mesenchyme cells initially seem to cover the amnion as well [11,12], but whether these cells are later replaced by mesodermal cells formed during gastrulation remains to be established [9].

## 2. Two types of amniogenesis: folding and cavitation

In amniotes, the amnion is the membrane that directly surrounds the embryo (figure 1*a*). It is avascular, elastic and contains the amniotic fluid that accumulates during the period of gestation in the amniotic cavity. The amniotic fluid contains embryonic waste, protects the embryo from dehydration, allows the embryo/fetus to move and functions as a ‘shock absorber’ [13].

Much of what we know on amnion development comes from studies in chicken, which undergo amniogenesis by folding (figure 1*b*). Here, the formation of the amnion starts anteriorly around the time the embryo has already formed 9–10 somite pairs (Hamburger–Hamilton stage 10) with the formation of an anterior amniochorionic fold [14]. This fold first envelops the head while advancing laterally in a cranial–caudal direction [15]. However, the enclosure of the head depends on the depression it makes in the proamnion (extraembryonic endoderm and ectoderm) while the embryo is flexing and extending [16]. Similarly, the caudal amniochorionic fold envelops the caudal part of the embryo and the amniochorionic folds meet dorsally at the midline, closing at around Hamburger–Hamilton stage 18, separating the amnion from the chorion/serosa. During closure, the cells in the amniochorion folds (of chicken and crocodile) align to form concentric lines of tension, with different elastic properties [17].

Oviparous amniotes undergo amniogenesis by folding; the amnion and chorion/serosa form and separate early, as they protect the embryo not only from desiccation, but also from adhesion to the eggshell [18]. In this regard, the amnion of oviparous amniotes such as snakes, lizards, chameleons and birds exhibits spontaneous rhythmic contractions. Indeed, the amnion retains its contractile capacity even after being isolated, suggesting it has smooth muscle-like characteristics. Depending on the species, the frequency of contractions is approximately 0.4/min for coldblooded animals and approximately 25/min for birds [19,20]. It has been hypothesized that the amnion of oviparous amniotes contracts to prevent the amnion from adhering to the eggshell [18]. These contractions would be autonomous, similar to the contractions of the intestine, for instance, that continue after removal from the animal. In eutherian mammals, extraembryonic motor activity has also been observed, but indirectly, resulting from contractions of the uterus.

**Figure 2.**
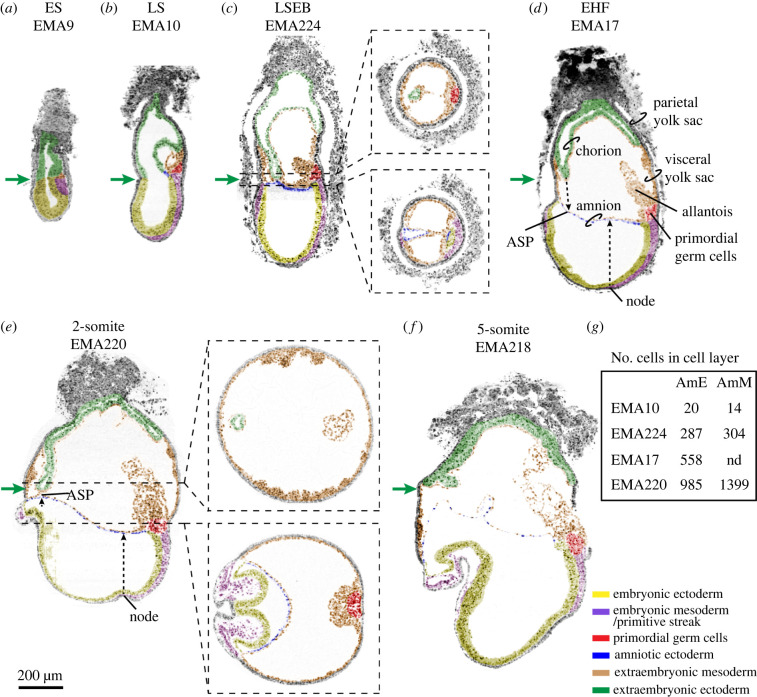
Gastrulation in the mouse. Mouse digital histological sections extracted from eMAP [22] and coordinates of sections are provided in the electronic supplementary material, table S5. Embryos used were (*a*) EMA9, ES, (*b*) EMA10, LS, (*c*) EMA224, LSEB, (*d*) EMA17, EHF, (*e*) EMA220, 2-somite stage and (*f*) EMA218, 5-somite stage. The sections were digitally painted to highlight different tissues of interest. Green arrows are aligned and show the part of the extraembryonic ectoderm at or nearest to the anterior. Dashed lines in the sagittal sections of EMA224 and EMA220 represent the place in the embryo where the transverse sections were extracted. In (*d*), the main features of the mouse embryo are indicated. In (*d*,*e*), dashed black arrows depict the position in the amnion of the projected node and anterior separation point (ASP). (*f*) Represents the total number of amniotic ectoderm and amniotic mesoderm cells present in the three-dimensional reconstruction of the depicted embryos (counting of EMA:224 after using MAPaint on high resolution, with stacked grey level images to make reconstructions). ASP: anterior separation point; EHF: early headfold; ES: early streak; LS: late streak; LSEB: late streak, early allantoic bud stage. Scale bar for all images is 200 µm.

Marsupial mammals and several eutherian mammals, such as ungulates (e.g. pig), most carnivores (e.g. dog) and some rodents (squirrel), have blastocyst embryos with an exteriorized epiblast. They typically exhibit amniogenesis by folding [21], similar to what is observed in chicken. Interestingly, humans, several non-human primates, some bats and some rodents (guinea pig and hedgehog) that have blastocyst embryos with an internalized inner cell mass/epiblast exhibit amniogenesis by cavitation (figure 1*b*) [21]. It can be hypothesized that the position of the inner cells mass determines the type of amnion formation. However, rodents such as the mouse that also exhibit an internalized inner cell mass show amniogenesis by folding (figures 1*b* and 2). In fact, mouse embryos form a single posterior amniochorionic fold during gastrulation (figures 1*b*, 2*b* and 2*c*) [6].

During the cavitation process, a proamniotic cavity is formed in the apical–basal polarized epiblast, forming an embryonic sac with dorsally squamous amniotic ectoderm and ventrally columnar epiblast. In human and non-primate embryos, it has recently been demonstrated that the amniotic ectoderm instructs the onset of gastrulation in posterior epiblast via BMP4 signalling [23,24].

It has been proposed that a separation is needed between the trophectoderm and epiblast to prevent precocious gastrulation induced by the trophectoderm, which could be why the epiblast cavitates in primates and why the polar trophoblast, known as Rauber's layer, disappears before gastrulation in species such as rabbits, pigs and cattle [25].

## 3. Formation of the amnion during gastrulation in mice

Just before gastrulation, the prestreak (PS) mouse embryo consists of a cup-shaped epiblast contacting proximally with an inverted cup-shaped extraembryonic ectoderm, both surrounded by a layer of visceral endoderm. At embryonic day (E)6.5 in early streak (ES) stage embryos, a continuous cavity, the proamniotic cavity, is visible between the epiblast and extraembryonic ectoderm (figure 2*a*). The dimensions of this cavity at the junction between the proximal epiblast and extraembryonic ectoderm are approximately 20–50 µm (anterior–posterior axis) by about 80–100 µm (left–right axis) [26–28]. Interestingly, the region of the proamniotic cavity associated with the lateral walls of the extraembryonic ectoderm-cup remains essentially unchanged regarding shape and dimensions during gastrulation, giving rise to a narrow tube, the proamniotic canal visible from late-streak stage (LS) until 2-somite stage (figure 2) [6,29].

Gastrulation initiates around E6.5 at the junction between epiblast and extraembryonic ectoderm in the proximal–posterior part of the mouse embryo. There, the primitive streak emerges (figure 2*a*) and two ‘wings’ of embryonic mesoderm migrate laterally (left and right) and distally towards the ‘bottom’ of the cup-shaped embryo, in between definitive ectoderm and definitive endoderm. Moreover, extraembryonic mesoderm emerges at the posterior end of the primitive streak and expands between extraembryonic ectoderm and visceral endoderm (figure 2*b,c*). As gastrulation progresses, the proportions between the anterior–posterior and left–right axes change and the anterior–posterior becomes the longer axis [26,27]. Subsequently, the embryo and its associated proamniotic cavity grow fast, whereas the dimensions of the base of the proamniotic canal remain relatively constant (green arrows in figure 2). The extraembryonic mesoderm expands and shows small cavities that ultimately form one single cavity, the extraembryonic coelom or exocoelom [6].

The differential growth between the rather static proamniotic canal and the fast-growing gastrulating embryo with its associated bulging cavities results in the generation of the amniochorionic fold in the posterior part of the embryo (figure 2*b*,*c*). Squamous epiblast-derived amniotic ectoderm delaminates from the proximal–posterior epiblast cells in contact with the extraembryonic mesoderm, together forming the amnion. The part of the amniochorionic fold consisting of cuboidal extraembryonic ectoderm and extraembryonic mesoderm will form the chorion. The amniochorionic fold and the enclosed exocoelom further expand (figure 2*c*). Eventually, the extraembryonic ectoderm and the amniotic ectoderm pinch off at the anterior separation point (ASP), sealing the proamniotic canal and splitting the proamniotic cavity into two separate cavities, the amniotic cavity lined by epiblast-derived cells and the ectoplacental cavity lined by extraembryonic ectoderm-derived cells (figure 2*d–f*). The expansion of the exocoelom creates a closed compartment of visceral yolk sac between the amnion and the chorion [6] (figure 2*d*–*f*).

**Figure 3.**
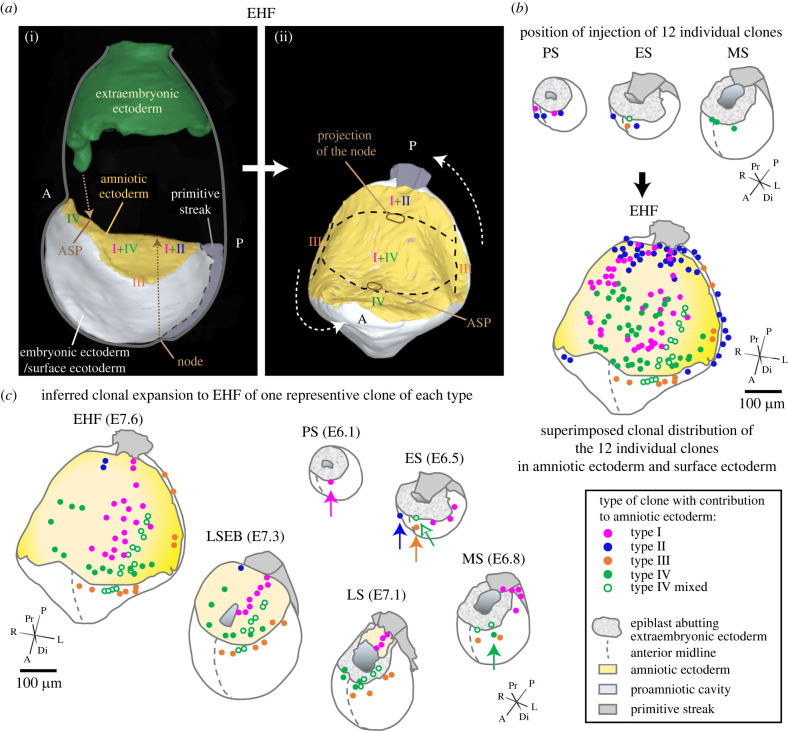
Amniotic ectoderm establishment and expansion in the mouse. (*a*) (i) shows a lateral three-dimensional view of a reconstructed EHF stage embryo (EMA17, eMAP) [22] (only the amniotic, embryonic and extraembryonic ectoderm and the primitive streak are shown); (ii) shows a rotated and tilted dorsal view of the amniotic and embryonic ectoderm and the primitive streak. Descendants of the four clone types that contributed to amniotic ectoderm establish different regions of the amnion. The projection of the node and the ASP mark along the anteroposterior axis domain borders. (*b*) Initial injection position in epiblast of 12 clones that contribute to amniotic ectoderm (top) and final distribution of the respective clone descendants after amnion/chorion separation (bottom) at EHF. All clone types except type IV contribute only a minority of descendants to the amniotic ectoderm, the majority of their descendants passing through the primitive streak and into the extraembryonic mesoderm or remaining as surface ectoderm (type III) (not presented; see table 1 in [30]). (*c*) Inferred expansion of one representative of clone type I to IV, and one new type IV mixed clone that also contributed to surface ectoderm. Injected clones are indicated by arrows. The intermediate plotted positions in ES, LS and LSEB stages are speculative, but based on the expansion of epiblast from anterior to posterior [31], the final position of clone descendants at the EHF end stage, and clonal histories [30]. The rotated and tilted embryo shapes in (*b,c*) show only epiblast/embryonic ectoderm, primitive streak and amniotic ectoderm. Embryo shapes were derived from three-dimensional views of reconstructed embryos for PS, ES, MS, LS and LPHF/EHF stages (eMAP) [22] and interpolations for MS and LSEB/EPHF based on external measurements of intact embryos and measurements over the amnion in EMA 224 (LSEB/EPHF) (eMAP) [22]. The clones are from [30] (type 1: #1, type II: #15, type III: #18, type IV: #27) except for the type IV mixed clone that unlike other amniotic ectoderm restricted type IV clones also contributes to surface ectoderm (unpublished, K.A.L, see electronic supplementary material, table S1). ASP: anterior separation point; E: embryonic day; EHF: early headfold; ES: early streak; MS: midstreak; LS: late streak; LSEB: late streak, early allantoic bud; PS: prestreak.

At E7.5–8.0, the mouse embryo initiates neurogenesis and somitogenesis, and the amnion stretches and extends especially laterally (left and right) to form a saddle-like shape that joins the ventrolateral contours of the surface ectoderm of the elongating embryo (figure 3*a*). At the 5-somite stage, the avascular amnion no longer has the appearance of a double-layered membrane of amniotic ectoderm facing the amniotic cavity and extraembryonic mesoderm the extraembryonic coelom, but the two cell layers appear intercalated (figure 2*f*) and embedded in the extracellular matrix of the basement membrane. At the 8-somite stage, the midgut endoderm of the embryo is still facing outwards, but around the 9–10 somite stage, the mouse embryo undergoes an 180^o^ axial rotation (dorsal–ventral) and acquires the typical fetal-position shape. The midgut becomes internalized and the two extraembryonic membranes, the amnion and visceral yolk sac, surround the entire embryo instead of being positioned dorsally from the embryo [10].

## 4. Lineage tracing of the amniotic ectoderm in mice

Single-cell labelling by iontophoretic injection of prestreak (PS) to midstreak (MS) mouse embryos followed by embryo culture for several days has enabled the construction of fine-resolution fate maps of labelled-cell descendants with known spatial and temporal origin [32]. Such an approach was used to establish an amniotic ectoderm fate map to trace and track the cells that form this cell layer (figure 3). Cells were labelled in PS to MS embryos followed by embryo culture up to the 5 somites stage [30]. Progenitors of all labelled clones contributing to the amniotic ectoderm were confined to a triangular area in the proximal–anterior region of the embryo, maximal 50 µm distal from the extraembryonic–embryonic junction at the midline and 50 µm lateral from the anterior midline.

Subsequent lineage analysis resulted in the identification of four different types of clonal behaviour (types I–IV) (figure 3*a*) [30]. The type I and IV clones that establish the bulk of the amniotic ectoderm both originate in the extreme proximal–anterior epiblast, less than 20 µm from the embryonic–extraembryonic junction of PS and early streak (ES) stage embryos (figure 3*b*) [30]. Despite their common origin, both types of clone descendants take opposite routes.

Type I clones expand posteriorly in PS and ES stage embryos (figure 3*b*). Part of this early population initiates the formation of the amniotic ectoderm at MS/late streak (LS) at the posterior end of the primitive streak; this is the founding population (figure 3*b*,*c*). The other part of the type I clone descendants contribute to the extraembryonic mesoderm, and sometimes to primordial germ cells (PGCs), via the primitive streak [30]. Conversely, descendants of the type IV clones remain anteriorly and are lineage restricted to the amniotic ectoderm from early MS stage onwards. They probably enter the amniotic territory at LS stage (figure 3*c*; electronic supplementary material, table S1). Type I and type IV clones together form the main body of the amniotic ectoderm until closing at the ASP.

Initiation of amniotic ectoderm formation opposite to the anterior region, at the proximal–posterior side of the embryo, may seem counterintuitive. However, the circumference of the extraembryonic–embryonic junctional zone only increases by about 12% between ES to LS stage embryos. Hence, descendants of the anteriorly positioned type I and II clones only have to bridge a short distance to the posterior side (figures 2*a*,*b* and 3*c*).

While type I and IV clones form the main body of the amniotic ectoderm, the type II and III clones then contribute small number of descendants, respectively, to the posterior and lateral peripheral regions [30] (figure 3).

A recent prospective cell fate map study generated from spatially resolved transcriptomics on defined areas of mouse embryos between E5.5 and E7.5 [33] concluded that the progenitors of amniotic ectoderm are also located in the extreme proximal–anterior epiblast.

## 5. Amnion expansion and closure

The scaling between early amniotic ectoderm and axial embryonic expansion, both initiating posteriorly and shortly followed by an anterior expansion, is remarkable. Based on nuclei counting in digitally painted cells in images of reconstructed embryos of the eMouse Atlas Project (eMAP) [22], the amniotic ectoderm primordium consists of approximately 20 cells at the LS, about five lines of four cells (EMA10) (figure 2*g*). At the late-streak early bud stage (LSEB), the amnion approaches anterior continuity and exhibits a dramatic increase in surface size and cell number [30] (figures 2*g* and 3*c*). About 287 cells are present in amniotic ectoderm and about 304 cells in amniotic mesoderm. The clonal analysis shows that the type IV clones contribute significantly to this expansion, and that type I and IV descendants meet from opposing directions. Although the expansion of type I and type IV together is sufficient to account for the increase in cell number in the amniotic ectoderm, additional cells begin to be recruited peripherally (type III) and posteriorly (type II) (figure 3*b*; electronic supplementary material, table S1). At this stage, the turgor in the exocoelomic and amniotic cavity is increasing, and the ventrolateral morphogenesis of the embryo initiates. Hence, it is not unexpected that type II and III clone descendants contribute to the lateral parts of the amniotic ectoderm or border of the surface ectoderm mainly during this period (figure 3*b*; electronic supplementary material, table S1).

Consistently, the posterior border of type IV descendants never crosses the projection of the node on the amnion [30] (figure 3*a*,*b*), showing that expansion of the amniotic ectoderm catches up from anteriorly and that type I and IV descendants overlap in the segment between the former ASP and the projection of the node. How this ‘contact’ and ‘crossing’ is regulated is unclear.

The left–right diameter at the base of the proamniotic canal hardly increases between early-streak and LSEB (figure 3), despite considerable extraembryonic and embryonic growth and morphogenesis. The stabilizing mechanism is unclear. The expansion of the visceral yolk sac cavity is likely to cause vertical ‘stretching’ at the presumptive ASP, which would apply a centripetal force on the amniotic tissues, drawing the open edges together and driving closure of the canal. There is, however, little indication of centripetal forces in the cell arrangement within type I and type IV clones (figure 3), as clones extend as lines of cells aligned in an anteroposterior direction. Transient cell rearrangement at closure involves only a limited number of very squamous and stretched cells in the immediate vicinity of the ASP (EMA322). The event can therefore easily be missed, especially with the endpoint timing of this clonal analysis. Nonetheless, it is remarkable that the type IV clone (fig. 3*a* in [30]) and identifiable in figure 3*b* can expand through the closing area without any trace of disturbance of anteroposterior expansion.

The developmental challenge for the mouse embryo is how the proamniotic cavity, which is an expanding hole, can be spanned from its edges; the evolutionary success is in the timing: managing to get the job done just before significant axial extension begins anterior to the node, when an intact amnion is required just to keep up with the pace.

## 6. Molecular signature of the cells of the amnion in mouse

Recently, a strong collective effort has been made to characterize the molecular signature of mouse embryos during gastrulation (between E6.5 and E8.5) using single-cell transcriptomics [34,35]. These efforts have generated comprehensive datasets that have been explored to understand the molecular signatures and developmental trajectories of many embryonic tissues. However, the development of the amniotic ectoderm and mesoderm has not been contemplated. Therefore, we have explored one of the two datasets available online [35] for the presence of amniotic ectoderm and mesoderm cell populations.

**Figure 4.**
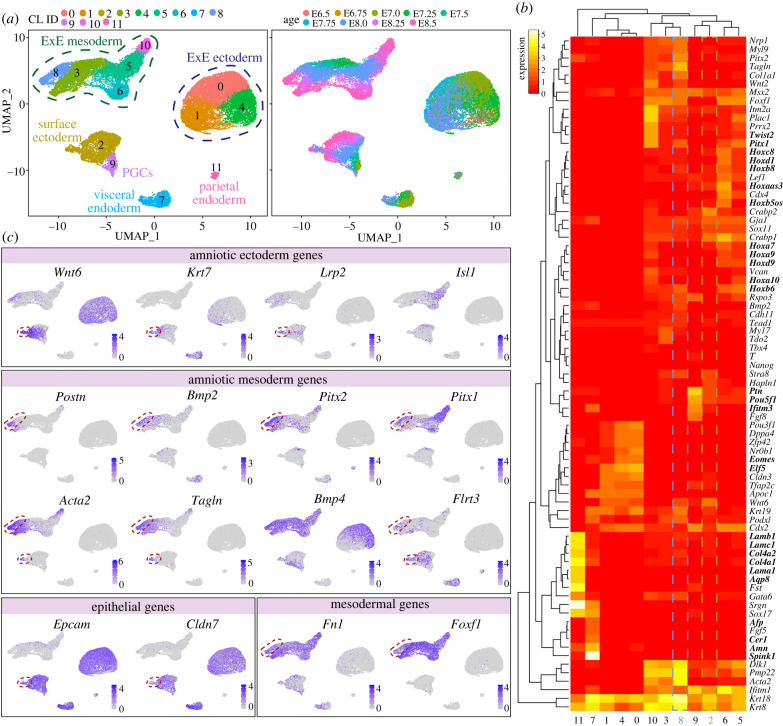
Identification of the molecular signature of the amniotic ectoderm and mesoderm in mice. (*a*) UMAP plots showing 12 clusters of cells; (i) shows the originally given signature [35] and (ii) is coloured by embryonic age (E). The cluster identity (CL) assigned to each of the clusters reflects its number of cells, 0 being the largest. (*b*) Heatmap depicting several differential gene expression sets of interest. (*c*) Expression of selected genes on the UMAP plot.

We have extracted several identified sub-populations (surface ectoderm, extraembryonic mesoderm and mesenchyme) that could include our populations of interest, plus several other clusters corresponding to additional extraembryonic populations (allantois, extraembryonic ectoderm, parietal endoderm, visceral endoderm and PGCs) for comparison. After a standard quality control step, we performed cell clustering analysis using a Seurat-based workflow [36]. Using uniform manifold approximation and projection (UMAP) analysis, we identified 12 clusters corresponding to the previously identified sub-populations (extraembryonic ectoderm, parietal endoderm, visceral endoderm, PGCs and surface ectoderm) (figure 4*a*). We also plotted the embryonic age and observed that the extraembryonic ectoderm, parietal endoderm and visceral endoderm were not well represented at E8.25–E8.5, but the extraembryonic mesoderm and surface ectoderm show cells of all ages (figure 4*a*). Interestingly, the population of extraembryonic mesoderm/mesenchyme was separated into five clusters.

We next calculated the differentially expressed genes in each of the obtained clusters compared to the other clusters [p_val_adj < 0.05 and pct.1 (proportion of cells showing expression per cluster) > 0.6] (electronic supplementary material, table S2) and provide a heatmap of some of the most relevant ones (figure 4*b*). The parietal endoderm (cluster 11) expressed several laminins and collagens, but also *Aqp8*, suggesting the involvement of the parietal endoderm in water transport. The visceral endoderm (cluster 7) expressed known markers such as *Cer1*, *Afp* and *Amn*, but also high levels of trypsin inhibitor *Spink1*. The three clusters of extraembryonic ectoderm (clusters 0, 1 and 4) showed expression of expected markers, such as *Eomes* and *Elf5*. The cluster of PGCs (cluster 9) showed expression of *Pou5f1* and *Ifitm3* and specific high levels of *Ptn*, encoding a heparin-binding growth factor not previously associated with mouse PGCs, but shown to have a mitogenic effect in PGCs in pig [37]. From the five clusters of extraembryonic mesoderm (clusters 3, 5, 6, 8 and 10), cluster 5 and cluster 6 expressed many different *Hox* genes, suggestive of the base of the allantois, whereas cluster 10 showed high-specific levels of *Pitx1* and *Twist2* present in the tip of the extending allantois [38].

Exploring the expression of the few known markers of amniotic ectoderm, such as *Wnt6* and *Lrp2* [38] (figure 4*c*), suggested that the population of amniotic ectoderm may be contained within cluster 2 (surface ectoderm). This subpopulation of cluster 2 was also positive for *Krt7*. At the developmental stage analysed (E6.5–E8.5), the cells in the surface ectoderm and amniotic ectoderm may not be sufficiently different to partition into separate clusters. Notably, a strong similarity between surface ectoderm and amniotic ectoderm has also been recently reported in a unique human embryo of 16–19 days post-fertilization analysed by single-cell transcriptomics [39]. We also plotted the expression of *Isl1*, a marker recently associated with amniotic ectoderm in primates and in differentiated human amnion ectoderm-like cells [23]. However, we observed reduced levels of *Isl1* in the *Wnt6* + *Krt7* + *Lrp2* + population.

Plotting the expression of amniotic mesoderm markers such as *Postn* [40] and *Bmp2* [41] (figure 4*c*) indicated that the cluster 8 may correspond to the amniotic mesoderm. Other markers highly expressed in this cluster were *Pitx1* and *Pitx2* (figure 4*c*)*.* Interestingly, smooth muscle cells markers such as *Tagln* and *Acta2* were not only highly expressed in amniotic mesoderm (figure 4*c*), but also in the population of *Wnt6* + *Krt7* + *Lrp2* + cells, suggesting that the amniotic ectoderm may undergo some degree of epithelial-to-mesenchymal transition. Furthermore, we observed high expression of *Bmp4* in extraembryonic mesoderm (figure 4*c*), in agreement with our previous observations of Bmp signalling activity in that tissue [40]. Finally, other markers that may be useful to differentiate between amniotic ectoderm and amniotic mesoderm are epithelial markers *Epcam* and *Cldn7* and mesenchymal markers *Fn1* and *Foxf1* [38]. Notably, genetic defects in both *Fn1* and *Foxf1* lead to pronounced defects in extraembryonic mesoderm [42,43]. Whereas the deletion of *Foxf1* leads to a small and inflexible (closed) amnion among other defects, the absence of *Fn1* resulted in several defects in mesodermal tissues and although there was a undersized closed amnion, the amniotic cavity seemed to lack pressure [42,43].

**Figure 5.**
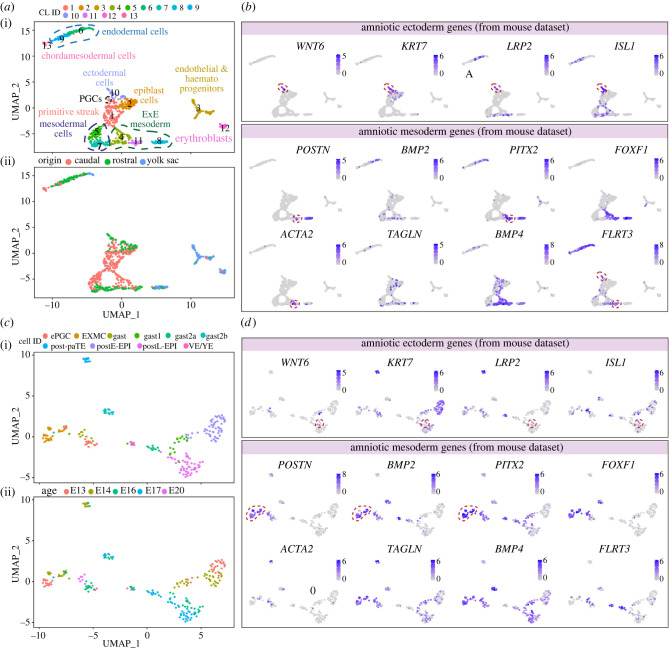
Expression of markers of amniotic ectoderm and mesoderm identified in mice, in human and cynomolgus monkey gastrulating embryos. (*a*) UMAP plots showing 14 clusters (CL) of cells of a single human embryo of 16–18 days post-fertilization; (i) shows the original clusters [39] and (ii) is coloured by embryo part (embryo was divided into three parts). (*b*) Expression of selected genes of interest on the UMAP plot. (*c*) UMAP plots showing 10 clusters of cells combining several cynomolgus monkey embryos [12,44]; (i) shows the original clusters and (ii) is coloured by embryonic age (E) in days post-fertilization. (*d*) Expression of selected genes of interest on the UMAP plot. ePGC: early PGC; EXE: extraembryonic; EXMC: exocoelomic membrane; gast: gastrulating cells; post-paTE: postimplantation parietal trophectoderm; postE-EPI: postimplantation early epiblast; postL-EPI: postimplantation late epiblast; VE/YE: visceral endoderm/yolk sac endoderm.

Although the amnion in primates and the amnion in mice forms via different mechanisms (cavitation versus folding, respectively), we have plotted available single-cell datasets from gastrulating human [39] and cynomolgus monkey [12,44] embryos, using the original cluster annotation (figure 5) and we provide the differentially expressed genes (electronic supplementary material, tables S3 and S4). Interestingly, when interrogating the expression of several genes representative of amniotic ectoderm in mice (*WNT6*, *KRT7* and *LRP2*), they were expressed in *ISL1* + amniotic ectoderm cells in human (figure 5*b*), but to a lesser extent in monkey probably owing to the reduced amount of cells or absence of that cell population of interest in the dataset (figure 5*d*). As in human, amniotic ectoderm in monkey has been shown to express high levels of ISL1 [23]. Moreover, genes representative of the amniotic mesoderm in mice (*POSTN* and *PITX2*) were expressed in a specific cluster of extraembryonic mesoderm in both human (cluster 11) (figure 5*b*) and monkey (cluster EXMC) (figure 5*d*), suggesting a degree of similarity regarding the molecular signature of these two cell types between mice, monkey and human.

## 7. Conclusion/future perspectives

The amnion remains an elusive tissue. Its small size and its well-hidden development within the mouse conceptus hampers studying its early development. Review of clonal analysis data provides, however, a comprehensive view on the origin and growth of amniotic ectoderm cells. Recently, several *in vitro* models using human pluripotent stem cells have sparked debate as it remains unclear whether human pluripotent stem cells are capable of differentiating into trophoblast, suggesting totipotency, or whether that is actually bona fide amniotic ectoderm that has been differentiated [45,46]. This uncertainty comes from the lack of a molecular signature of *in vivo* human trophectoderm and amniotic ectoderm at the time of implantation. However, our knowledge on gene expression in early human amnion is currently restricted to one human embryo of 16–19 days post-fertilization recently used for single-cell transcriptomics [39]. The molecular signature of amniotic mesoderm was not determined in that study. Although several datasets of single-cell transcriptomics, each containing thousands of cells, of gastrulating embryos exist in the mouse, little attention has been dedicated to amnion development.

In contrast with mouse, the primate amnion has recently been gaining more attention. Interestingly, several studies on early primate embryogenesis and stem cell-based embryonic models have shown that the amnion in primates may act as signalling centre for normal embryonic development [23,24,47–49]. Moreover, the primate amnion has also been shown as the origin of PGCs [44]. Hence, broadening our knowledge of amniogenesis in amniotes will contribute to our understanding of pluripotency and early lineage restriction, and may help in clarifying amnion defects.

## Data Availability

All data are provided in the manuscript or the electronic supplementary material [50], or referenced in the text.
